# Maximising Embedded Pharmacists in AGed CAre Medication Advisory Committees (MEGA-MAC): protocol for implementing Australia’s new guiding principles for medication management in residential aged care facilities using knowledge brokers and a national quality improvement collaborative

**DOI:** 10.1186/s13012-025-01449-0

**Published:** 2025-08-04

**Authors:** Amanda J. Cross, Brooke Blakeley, Kate Laver, Terry P. Haines, Sarah N. Hilmer, Atish Manek, Alexandra Bennett, Angelita Martini, Lyntara Quirke, Mary Ann Kulh, Sara L. Whittaker, Dayna R. Cenin, Anthony Hobbs, Joanne Money, Karina Rieniets, Kris Salisbury, Alene Sze Jing Yong, J. Simon Bell

**Affiliations:** 1 Centre for Medicine Use and Safety, Faculty of Pharmacy and Pharmaceutical Sciences, Monash University, Parkville Campus, Parkville, VIC 3052 Australia; 2 College of Nursing and Health Sciences, Flinders University, Bedford Park, South Australia Australia; 3 School of Primary and Allied Health Care, Faculty of Medicine Nursing and Health Sciences, Monash University, Clayton, VIC Australia; 4 Kolling Institute, Northern Sydney Local Health District and The University of Sydney, Sydney, NSW Australia; 5 Departments of Clinical Pharmacology and Aged Care, Royal North Shore Hospital, Sydney, NSW Australia; 6 Department of General Practice, Faculty of Medicine Nursing and Health Sciences, Monash University, Clayton, VIC Australia; 7 New South Wales Therapeutic Advisory Group, Sydney, NSW Australia; 8 Calvary Health Care, Sydney, NSW Australia; 9 School of Allied Health, The University of Western Australia, Crawley, WA Australia; 10 Consumer Representative, Australian Capital Territory, Scullin, Dementia Australia Australia; 11 School of Medicine and Psychology, Australian National University, Australian Capital Territory, Acton, Australia; 12 Rehabilitation, Ageing and Independent Living (RAIL) Research Centre, School of Primary and Allied Health Care, Monash University, Frankston, VIC Australia; 13 Brightwater Research Centre, Brightwater Care Group, Inglewood, WA Australia; 14 School of Population and Global Health, University of Western Australia, Perth, WA Australia; 15 Grampians Health, Ballarat, VIC Australia; 16 Faculty of Medicine and Health, School of Pharmacy, The University of Sydney, Sydney, NSW Australia

**Keywords:** Long-term care, Implementation science, Knowledge translation, Practice guidelines as a topic, Quality use of medicines, Quality improvement, Quality indicators, Clinical governance, Interrupted time series analysis, Net benefit analysis

## Abstract

**Background:**

Incomplete or delayed implementation of Guidelines can lead to potentially avoidable medication-related harm. All Australian residential aged care facilities (RACFs) are recommended to have access to a multidisciplinary medication advisory committee (MAC) to provide clinical governance of medication management. The objective of this trial is to evaluate the effectiveness and relative net benefit of using knowledge brokers, supported by a national quality improvement collaborative, to implement Australia’s new Guiding Principles for Medication Management in Residential Aged Care Facilities (Guiding Principles).

**Methods:**

The Maximising Embedded Pharmacists in AGed CAre Medication Advisory Committees (MEGA-MAC) trial will be conducted in partnership with RACFs operated by three aged care provider organizations across four states of Australia. The intervention will involve knowledge broker dyads (pharmacist plus a MAC representative [e.g. nurse]) developing, implementing and evaluating RACF-specific local action plans to implement the Guiding Principles in up to 15 RACFs. Knowledge broker dyads will be supported by a national quality improvement collaborative (MEGA-MAC collaborative) comprising clinical experts, implementation scientists and resident and caregiver representatives. An interrupted time series design will be used to assess change over time with three pre-intervention (-6, -3 and 0 months) and three post-intervention assessment time points (+ 3, + 6, + 9 months). The primary outcome will be change in pre/post RACF-level concordance with the Guiding Principles measured using quality indicators (score 0 to 28, higher scores = greater concordance). A net benefit analysis will be conducted to examine the relative costs and benefits of implementing the intervention.

**Discussion:**

The MEGA-MAC trial investigates a novel multifactorial knowledge translation strategy to improve the uptake of guidelines and support safe and appropriate use of medication in RACFs. We anticipate that the findings will provide new information on the role of healthcare professionals as knowledge brokers, MACs, and quality improvement collaboratives for effective guideline implementation in RACFs.

**Ethics and dissemination:**

Ethics approval obtained from Monash University and Grampians Health Human Research Ethics Committees. Findings will be disseminated through professional and lay media, conference presentations and peer-reviewed publications.

**Trial registration:**

Australian New Zealand Clinical Trial Registry (ANZCTR): ACTRN12624000894594. Registered 22nd July 2024 – Prospectively registered. https://www.anzctr.org.au/ACTRN12624000894594.aspx

**Supplementary Information:**

The online version contains supplementary material available at 10.1186/s13012-025-01449-0.

Contributions to Literature • This trial is the first to evaluate the use of knowledge broker dyads and a quality improvement collaborative to improve concordance with guidelines in residential aged care facilities

• This trial is the first to evaluate the net-benefit of a knowledge broker intervention in residential aged care facilities.

• This trial will evaluate novel system-level roles for pharmacists and medication-advisory committee representatives.

• If effective, the knowledge translation strategy could be adapted to other therapeutic areas and settings, and evaluated to determine its effect on the safety and effectiveness of medications for older adults.

## Background

Medication safety in older people remains a global public health challenge. The World Health Organization (WHO) has recognized the importance of medication safety through its Medication without Harm Global Patient Safety Challenge [1]. However, poor implementation of clinical guidelines and evidence-based guideline recommendations remains a key barrier. The 2024 WHO Global Research Agenda on Knowledge Translation and Evidence-informed Policy Making identifies the need to improve efficiencies and synergies of knowledge translation in healthcare [2], and the WHO identifies governance and leadership as key factors required to strengthen residential care systems [1, 3].

In Australia, people living in residential care are prescribed an average of 10 regular medications, and over half are prescribed potentially inappropriate medications, which may result in adverse events or unplanned hospitalizations [4, 5]. There is up to fourfold variability across residential aged care facilities (RACFs) in nine of the 10 most prevalent medications, with this variation unlikely to be explained by clinical characteristics of residents alone [6]. This highlights the potential value of knowledge sharing and benchmarking across RACFs. In 2021 the Australian Commission on Safety and Quality in Health Care identified 10 priority action areas for residential aged care, including a need to improve the governance, composition and operation of Medication Advisory Committees (MACs) (priority 4) and a recommendation to trial the role and impact of onsite aged care pharmacists (priority 10) [7].

A MAC is a “multidisciplinary committee that provides overarching governance of medication management within a RACF to ensure the judicious, appropriate, safe and quality use of medicines” [8]. All RACFs in Australia are required to establish or have direct access to a MAC. Australian MACs share similarities with international quality circles (multidisciplinary peer review groups) that support primary healthcare professionals [9, 10]. as well as hospital-based drug and therapeutics committees [11]. Our previous work highlighted the importance of multidisciplinary MAC membership, scope for greater pharmacist involvement, and the need to leverage data (e.g. quality indicators) to inform quality improvement and practice [12]. It also identified that MACs often work in isolation and opportunities exist to share learnings, experiences and insights to promote best practice [12]. This work informed the User Guide: Role of a Medication Advisory Committee (User Guide) published in 2022 by the Australian Government’s Department of Health and Aged Care [8]. However, a national survey of MACs conducted in 2023–24 revealed there is considerable variability in the structure and function of MACs, 58% of MACs reported their membership was multidisciplinary (medical, nursing and pharmacy) and 28% of MACs reported that they performed all recommended functions related to quality improvement [13].

In November 2022, Australian Government’s Department of Health and Aged Care published updated Guiding Principles for Medication Management in Residential Aged Care Facilities (Guiding Principles) [14]. The Guiding Principles support RACFs to promote a systems approach to medication safety. In particular, the Guiding Principles recommend parameters and procedures for medication management within RACFs, with good clinical governance an important tool to support implementation and monitoring of these processes. Effective implementation of the Guiding Principles, including the User Guide, which is a supplementary resource to the Guiding Principles, requires a targeted knowledge translation strategy. There is emerging evidence on the role of knowledge brokers and quality improvement collaboratives in facilitating guideline implementation in practice [15, 16]. Knowledge brokers are individuals or groups that help to move knowledge from those who create the knowledge (e.g. guideline developers) to those that use the knowledge (e.g. health care professionals) [15]. A quality improvement collaborative is a structured, multi-site initiative where healthcare professionals work together, supported by experts and a shared framework, to implement best practices, improve clinical processes, and enhance patient outcomes [17]. Quality improvement collaboratives are a cost-effective way to develop knowledge, motivation and quality improvement capacity of individuals and health systems [18, 19]. An Australian study involving a national quality improvement collaborative for implementing Australia’s Clinical Practice Guidelines and Principles of Care for People Living with Dementia demonstrated 42.1% immediate increase in clinician self-reported adherence to the guideline recommendations [16, 20].

The objective of this trial is to evaluate the effectiveness and examine the relative net benefit of using knowledge brokers, supported by a national quality improvement collaborative, to implement the Guiding Principles.

## Methods

### Design

**Fig. 1:**
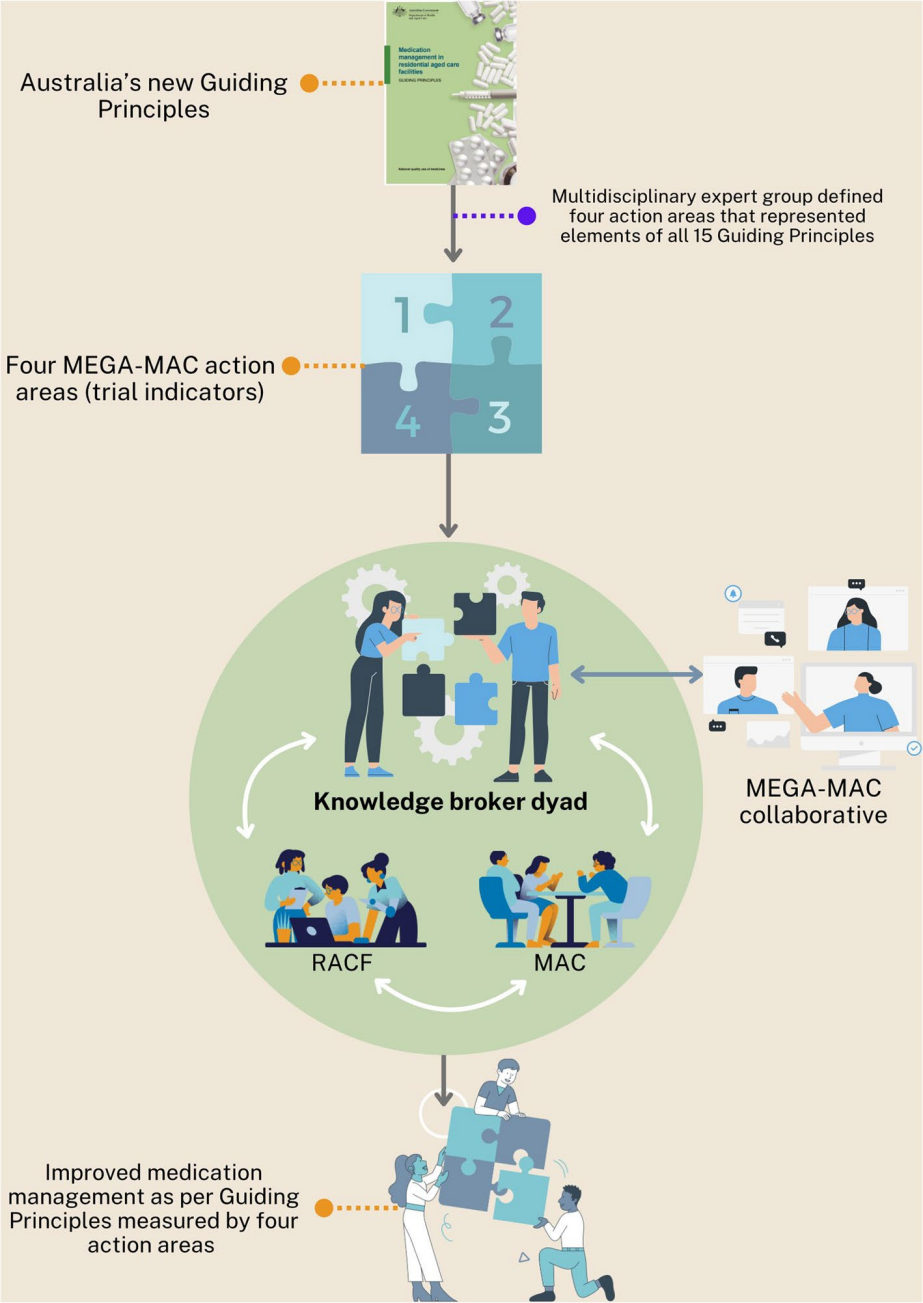
Overview of the MEGA-MAC intervention

The Maximising Embedded pharmacists in aGed cAre Medication Advisory Committees (MEGA-MAC) trial will use an interrupted time series design to investigate the effect of knowledge broker dyads (pharmacist and MAC representative) supported by a national quality improvement collaborative (MEGA-MAC collaborative) to implement the Guiding Principles (Fig. 1). This design will involve retrospective (pre-intervention) and prospective (post-intervention) data collection. This design was selected because the quality improvement collaborative involves interventions being delivered concurrently making alternate designs involving randomisation (e.g. stepped-wedge) less feasible. Interrupted time series analyses are considered a strong quasi experimental design for evaluating quality improvement initiatives and uptake of clinical practice guidelines [21, 22].

The trial was designed in partnership with the three participating aged care provider organizations and the following foundational activities:

- Online survey of 120 current MACs in Australia to explore structure, scope and function of MACs [13]. The survey was based on the Department of Health and Aged Care Audit tool and checklist for a Medication Advisory Committee [23] and the User Guide: Role of a Medication Advisory Committee [8].
- Semi-structured interviews with 20 MAC members in Australia to explore the current structure, scope and function of MACs.
- Systematic review of the roles and effectiveness of knowledge brokers in implementing clinical practice guidelines [15].
- 15 semi-structured interviews with early adopters of Australia’s embedded on-site aged care pharmacist model [24].
- Ongoing learnings from the Evidence-based Medication knowledge Brokers in Residential Aged CarE (EMBRACE) trial [25].

This trial protocol conforms to the Standard Protocol Items: Recommendations for Interventional Trials (SPIRIT) guidelines (Appendix 1) [26, 27]. The economic evaluation will be conducted alongside the trial and will be reported in accordance with the Consolidated Health Economics Evaluation Reporting Standards (CHEERS) checklist [28]. The trial was prospectively registered with the Australian and New Zealand Clinical Trials Registry (ANZCTR) [Registration #:ACTRN12624000894594. Date registered: 22/07/2024].

### Setting

The trial will involve up to 15 RACFs, and their MACs, operated by three aged care provider organisations in four Australian States (Victoria, New South Wales, South Australia, Western Australia). The three aged care provider organisations include one government and two not-for-profit organisations, and span across metropolitan, regional, rural and remote geographical regions. In Australia, RACFs provide care for individuals who’s care needs cannot be adequately met at home. Internationally, RACFs are often referred to as long-term care facilities or nursing homes [29, 30]. In Australian RACFs, medications are typically prescribed by visiting medical practitioners, dispensed by external community pharmacies, and administered by on-site nurses or credentialed aged care workers [31]. Some RACFs have their own MAC while other RACFs operate under the governance of an overarching MAC within a larger organisation. With an overarching MAC, the MAC should still identify, prioritise and address local medication-related issues at the individual RACF-level [8].

### Governance and engagement

The trial will be overseen by the Project Management Team comprising the lead investigators and Project Manager. The Project Management Team will meet with the aged care provider organisation representatives and knowledge broker dyads regularly throughout the study to monitor the data collection, conduct of the study and to identify and mitigate any potential risks.

The trial will be supported by a Project Interest Holder Group [32] comprising government agencies (Department of Health and Aged Care, Australian Commission on Safety and Quality in Health Care, Aged Care Quality and Safety Commission), professional groups (Australian and New Zealand Society for Geriatric Medicine, Royal Australian College of General Practitioners, Australian College of Nursing, Pharmaceutical Society of Australia, Pharmacy Guild of Australia), aged care provider organisations (Ageing Australia [formerly known as Aged and Community Care Providers Organisation]), primary health networks (Western Victoria Primary Health Network) and consumer and carer representatives (Older Persons Advocacy Network, Dementia Australia). The Project Interest Holder Group will meet every six months to provide overall strategic advice for the project, taking into consideration current and emerging practice issues. Additional meetings may be considered if needed. A Data Safety Monitoring Board will meet as and when required if potential or actual adverse events caused by the intervention are reported during the study. The Board will involve an independent group of three healthcare professionals with relevant clinical expertise (e.g. general practitioner, geriatrician, nurse, pharmacist).

### Recruitment, eligibility and consent of RACFs

The three aged care provider organisations’ leadership team will identify RACFs that are eligible and interested in participating. RACFs will be eligible if they have a MAC or commit to establishing a MAC by the commencement of the trial. Once eligible RACFs are identified at the aged care provider organisational level, a member of the Project Management Team will explain the trial to eligible RACFs, provide the explanatory statement and obtain written informed consent from the RACF manager or an authorised representative of the aged care provider organisation.

This will be a RACF-level intervention, individual residents will not be recruited to participate. Nurses, aged care workers, visiting pharmacists and medical practitioners at each RACF and MAC members will be made aware of the objectives of the trial and the nature of the intervention via letters, posters and notice boards. Residents and their families will be made aware of the trial through distribution of notices and posters at each RACF.

### Recruitment of knowledge broker dyad

The knowledge broker dyad will consist of a pharmacist and a MAC representative. Each aged care provider organisation will employ or contract their own knowledge broker dyad(s) for each participating RACF through a local expression of interest process. Use of a local knowledge broker is intended to build on existing relationships between the knowledge broker dyad and staff within each RACF, as introducing external knowledge brokers may be less effective [33]. The knowledge broker dyad will work at the RACF-level, and individual knowledge brokers may work across more than one RACF (e.g. a pharmacist may work across two RACFs and collaborate with a different MAC representative for each RACF).

Pharmacists will be eligible to be a knowledge broker if they are:

- Registered with the Australian Health Practitioner Regulation Agency (AHPRA)
- Available to commit to 0.1 full time equivalent per RACF for the 9-month intervention (approximately 4 h per week for the knowledge broker role and 0.5 h per week for trial-related tasks [e.g. attending trial meetings, documentation]); and
- Able to complete specific tasks of the research project, and all relevant aged care provider organisation induction and occupation, health and safety requirements.

Prior aged care experience and credentialing to complete medication management reviews or be an aged care on-site pharmacist will be desirable but not a requirement.

MAC representatives will be eligible to be a knowledge broker if they are the MAC chair or another suitable current MAC member for their RACF’s MAC, and if they are:

- Registered with AHPRA (e.g. registered nurse), or have a health professional background (e.g. management staff working in a non-clinical role).
- Available to commit to 1.5 h per week per RACF for the 9-month intervention period (approximately 1 h per week for the knowledge broker role and 0.5 h per week for trial-related tasks [e.g. attending trial meetings, documentation]); and
- Able to complete specific tasks of the research project.
- A non-pharmacist MAC representative will be desirable but not a requirement.

A member of the Project Management Team will meet with each potential dyad member (pharmacist and MAC representative), provide the explanatory statement and an opportunity to ask questions, and obtain written informed consent.

### Recruitment of the national quality improvement collaborative (MEGA-MAC collaborative)

The MEGA-MAC collaborative will comprise the knowledge broker dyads plus a multidisciplinary panel of clinical experts, implementation scientists, and resident and caregiver representatives. The clinical experts, implementation scientists and consumers will be identified via professional contacts of the Project Management Team or participating aged care provider organisations. Potential participants will be eligible to be a MEGA-MAC collaborative panel member if they are: registered with AHPRA (e.g. medication practitioner, pharmacist, registered nurse); working in implementation science; or identify as a resident or caregiver representative. The Project Management Team will provide potential MEGA-MAC collaborative panel members with the explanatory statement and obtain written informed consent from the MEGA-MAC collaborative panel member.

### Knowledge broker dyad training

Knowledge broker training will be a requirement for pharmacists and desirable for MAC representatives and MEGA-MAC collaborative panel members. This is because the knowledge broker pharmacist will be responsible for leading the delivery of the intervention. Training will involve a series of online education modules (approximately 4 h in total) developed for the MEGA-MAC trial and hosted on the Guroo Learning platform. Content related to knowledge translation and change management will be based on previous education developed for the national quality improvement collaborative for implementing Australia’s Clinical Practice Guidelines and Principles of Care for People Living with Dementia [20]. The self-paced learning in this trial will encourage participants to reflect on their own experience, apply learnings to case studies, and discuss the education material via a chat function. The education modules will cover the following topics:

1. Introduction into medication safety and the quality use of medications in RACFs, including introduction to the Guiding Principles and the role of MACs.
2. Knowledge translation and the role of a knowledge broker, including a focus on the practical application of knowledge translation and how to be an effective knowledge broker dyad.
3. Change management and local action plans, including barriers and enablers to change and guidance on how to develop local action plans.
4. MEGA-MAC trial design and trial indicators.

## Intervention

### Knowledge broker dyad

The intervention will involve the knowledge broker dyad implementing the Guiding Principles together with the RACF and MAC, with support from the MEGA-MAC collaborative. The knowledge broker dyad will act as a knowledge manager, capacity builder, and linking agent [15], with activities including:

- Knowledge manager: Distributing and raising awareness about the Guiding Principles; embedding and applying the Guiding Principles into local contexts, policies and procedures; and assessing barriers and enablers to implementation in the local context.
- Linkage agent: Facilitating collaboration between key onsite and off-site stakeholders (e.g. aged care provider management, quality improvement teams, medical practitioners, nurses, aged care workers, allied health, and community pharmacists); engaging with other knowledge broker dyads via the MEGA-MAC collaborative; and being a conduit between the Project Management Team and the RACF employed staff and visiting team members.
- Capacity builder: Developing staff knowledge and skills to facilitate and enable improvements in medication management in line with the Guiding Principles; conducting audits and providing feedback on concordance with the Guiding Principles using the MEGA-MAC trial indicators to inform development of quality improvement initiatives; and evaluating effectiveness of those quality improvement initiatives.

**Fig. 2:**
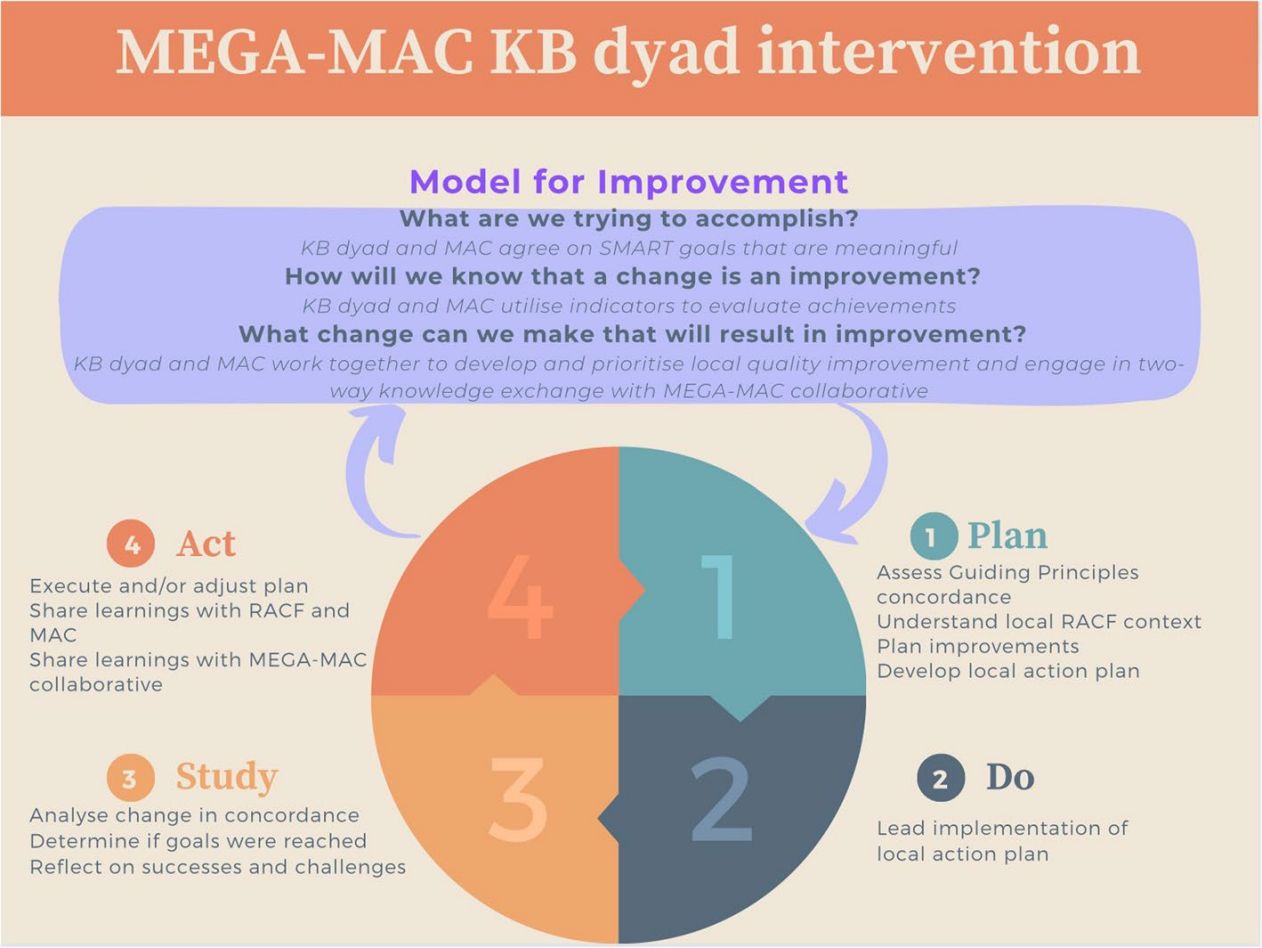
Description of the knowledge broker dyad intervention informed by the Institute of Health Improvement (IHI) model and the plan-do-study-act (PDSA) cycle. Abbreviations: KB, knowledge broker; MAC, medication advisory committee; RACF, residential aged care facility; SMART, specific, measurable, achievable, relevant, timely

The knowledge broker dyad will develop, implement, and evaluate RACF-specific local action plans quarterly based on each RACF's needs (Fig. 2). The local action plans will follow a systems approach using the United States Institute for Healthcare Improvement Model (IHI model) [34] and the Plan-Do-Study-Act (PDSA) cycle [35]. The knowledge broker dyad will use the three fundamental questions in the IHI model to identify why a change is needed, what improvement will look like and the changes that will result in improvement [34]. The PDSA cycle will be used as a systematic framework for continuous improvement in the real-world setting, including adapting planned actions according to ongoing feedback and monitoring [35]. The knowledge broker dyad will identify areas for improvement informed by a local context assessment, MEGA-MAC trial indicator results and with input from RACF staff, MAC members and the MEGA-MAC collaborative. Knowledge broker dyads will receive feedback on their local action plans from the Project Management Team, knowledge broker peers and the MEGA-MAC collaborative (Table 1).

**Table 1:** Knowledge broker dyad study activity requirements

Time point	Reporting and study activity requirement
−1-month time point	*Training and orientation:* Knowledge broker dyad will participate in RACF training/orientation and complete MEGA-MAC study training
Baseline (0-month time point)	*Local action plan:* Knowledge broker dyad will collaboratively develop local action plans, submit their local action plans for MEGA-MAC collaborative expert and peer review, provide feedback on their peers’ local action plans and present their local action plans at local MAC meetings *Intervention:* Delivery of knowledge broker dyad services as per local action plans and attendance at local MAC and MEGA-MAC meetings *Process evaluation:* Online survey with MEGA-MAC meeting attendees at the conclusion of MEGA-MAC meeting
3-month time point	*Local action plan:* Knowledge broker dyad will collaboratively reflect and revise local action plans, submit their local action plans for MEGA-MAC collaborative expert and peer review, provide feedback on peers’ local action plans and present their local action plans at local MAC meetings *Intervention:* Ongoing delivery of knowledge broker services as per local action plans and attendance at local MAC and MEGA-MAC meetings *Process evaluation:* Semi-structured interviews with knowledge broker dyads and online survey with MEGA-MAC meeting attendees
6-month time point	*Local action plan:* Knowledge broker dyad will collaboratively reflect and revise local action plans, submit their local action plans for MEGA-MAC collaborative expert and peer review, provide feedback on peers’ local action plans and present their local action plans at local MAC meetings *Intervention:* Ongoing delivery of knowledge broker services as per local action plans and attendance at local MAC and MEGA-MAC meetings *Process evaluation:* Semi-structured interviews with knowledge broker dyads and online survey with MEGA-MAC attendees
9-month time point	*Local action plan:* Knowledge broker dyad will reflect on success and challenges for previous quarter and present their findings at the local MAC and final MEGA-MAC meeting *Process evaluation:* Semi-structured interviews with knowledge broker dyads and online survey with MEGA-MAC attendees

*RACF* residential aged care facility, *MAC* medication advisory committee, *MEGA-MAC* national quality improvement collaborative

### MEGA-MAC collaborative

Knowledge broker dyads will be supported by the MEGA-MAC collaborative. This will be modelled on the national quality improvement collaborative for implementing Australia’s Clinical Practice Guidelines and Principles of Care for People Living with Dementia [20]. The MEGA-MAC collaborative will act as a real-time clinical network to enable sharing of experience and expertise between the knowledge broker dyads and support the knowledge broker dyads, RACFs, MACs and aged care provider organisations to implement the Guiding Principles and ensure quality use of medications.

**Fig. 3:**
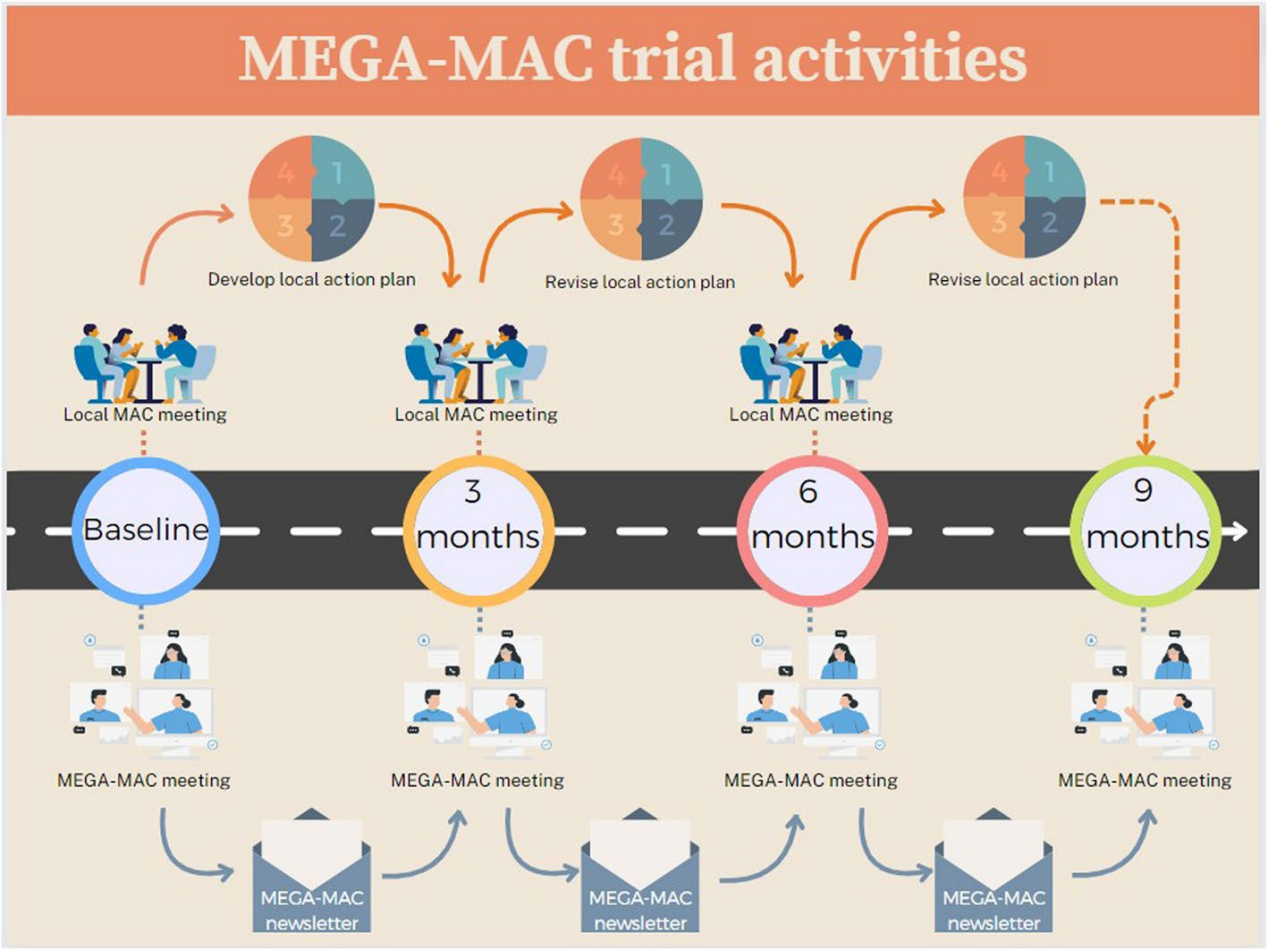
Visual representation of activities occurring during the MEGA-MAC study. Abbreviations: MAC, medication advisory committee; MEGA-MAC, national quality improvement collaborative

The MEGA-MAC collaborative will support the knowledge broker dyads through the following activities (Fig. 3):

- quarterly virtual ‘MEGA-MAC’ meetings (baseline, 3, 6 and 9 months);
- quarterly ‘MEGA-MAC’ newsletters (baseline, 3, and 6 months) that include trial updates and sharing of learnings;
- providing aggregate Guiding Principles concordance results from all participating RACFs that each knowledge broker dyad can use to benchmark against their RACF-level concordance;
- expert and peer review feedback on the knowledge broker dyads’ local action plans (baseline, 3 months and at 6 months); and
- ongoing ad-hoc expert and peer support via virtual meetings, and informal real-time peer support via SLACK (Searchable Log of All Communication and Knowledge) chat group.

Ongoing fidelity of the intervention delivered by the knowledge broker dyads will be monitored using the local action plans, the MEGA-MAC meetings and regular meetings with the Project Management Team.

### Data collection

Data will be collected from the participating RACFs at six timepoints (−6, −3, baseline, 3, 6, 9 months) by trained data collectors (aged care nurses, health or care professionals employed or contracted by each of the participating aged care provider organisations) (Table 2). Data collectors will attend an online training webinar on data collection led by the Project Management Team and will have access to data collection manuals and recorded training webinars to ensure consistency of data collection. Pre-intervention data (−6, −3, 0 months) will be collected retrospectively at or before baseline. Post-intervention data (+ 3, + 6, and + 9) will be collected prospectively. Trial data will be collected using a series of standard data collection forms and managed using Research Electronic Data Capture (REDCap), an electronic data capture tool hosted and managed by Helix (Monash University). REDCap is a secure, web-based software platform designed to support data capture for research studies [36, 37]. Trial data will be stored for a minimum of 15 years after publication of result in accordance with Australian National Health and Medical Research council (NHMRC) and Monash University guidance for medical research involving clinical trials. The final dataset will only be accessible to the investigators and statistician or epidemiologist.

**Table 2:** SPIRIT diagram – schedule of enrolment, intervention and assessment for the MEGA-MAC study

	Study period							Data source
Timepoint (months)	Pre-intervention period		Enrolment	Baseline	Intervention period			
	−6	−3	*-t*	0	3	6	9	
RACF enrolment								
Eligibility screen			x					
Consent			x					
Intervention								
KB dyad recruitment			x					
MEGA-MAC collaborative panel recruitment			x					
KB training			x					
Intervention implementation				x	x	x	x	
Data collection								
RACF characteristics information				x				Clinical and administration records; knowledge broker dyads
MAC characteristics information				x			x	Clinical and administration records; knowledge broker dyads
KB dyad and MEGA-MAC collaborative panel demographic information			x					Knowledge brokers and MEGA-MAC members
RACF concordance with Guiding Principles	x	x		x	x	x	x	NQIP records; psychotropic medicines register; nursing progress notes; medical records; RACFs register of policies/procedures/guidelines; register of residents who have CMR by a pharmacist; register of resident admitted to the RACF; MAC meeting agenda/minutes/associated papers
RACF-level proportions of hospitalisations, resident QoL, consumer experience, medication incidents	x	x		x	x	x	x	NQIP records, RACF electronic risk management system
Knowledge broker dyad activities				x	x	x	x	Local action plans; semi-structured interviews, online MEGA-MAC meeting surveys
Economic evaluation – intervention costs							x	MEGA-MAC project budget; MEGA-MAC meeting agenda/minutes/associated papers
Economic evaluation – downstream costs for the RACF				x			x	RACF staff
Economic evaluation – downstream costs for the RACF’s MAC	x	x		x	x	x	x	MAC meeting agenda/minutes/associated papers; MAC members

*CMR* comprehensive medication review, *KB* knowledge broker, *MAC* Medication Advisory Committee, *MEGA-MAC collaborative* national quality improvement collaborative, *NQIP* National Aged Care Mandatory Quality Indicator Program, *RACF* residential aged care facility, *-t* prior to baseline, *QoL* quality of life

### RACF-level concordance with Guiding Principles

The data collectors will assess RACF-level concordance with the Guideline Principles using a series of indicators developed for the MEGA-MAC trial by New South Wales Therapeutic Advisory Group (NSW TAG), in consultation with an expert advisory group and the Project Management Team [38]. The indicators will assess concordance with the Guiding Principles across four key domains: the RACF’s 1) MAC meeting; 2) policies, procedures and guidelines; 3) admission processes; and, 4) medication review processes (Tables 3, 4, 5, and 6). There will be 28 indicators across the four domains.

**Table 3:** Domain 1 Indicators and sub-indicators evaluating the RACF’s MAC meeting

Indicator	Numerator	Denominator	Target
Calculation of summary indicator			
Percentage of recommended medication governance activities undertaken by the RACF	Number of positive or NA responses to the listed RACF’s medication governance and management activities	9 (total number of responses)	High (towards 100%)
Sub-indicators regarding aspects of MAC structure or function		Possible responses	
1. The RACF’s MAC met in the last quarter		Yes/No	
2. The RACF’s MAC meeting was multidisciplinary		Yes/No	
3. When a MAC governs more than one RACF, a representative of this RACF was in attendance		Yes/No/NA*	
4. The RACF’s report of residents’ experiences from RACF residents, their carers, family and/or substitute decision-makers regarding medication-related issues was presented		Yes/No	
5. The RACF’s report of RACF storage of medicines was presented		Yes/No	
6. The RACF’s report of medication incidents was presented		Yes/No	
7. The RACF ‘s report of trends in the percentage (or numbers) of residents with polypharmacy was presented		Yes/No	
8. The RACF’s report of trends in the percentage (or numbers) of residents who received an antipsychotic medication was presented		Yes/No	
9. The RACF’s report of trends in the percentage (or numbers) of residents who received a comprehensive medication management review from an appropriately qualified pharmacist was presented		Yes/No	

^*^ NA is selected if the MAC governs one RACF only. One point is allocated for selecting N/A in response to this indicator

*MAC* medication advisory committee, *NA* not applicable, *RACF* residential aged care facility

**Table 4:** Domain 2 Indicators and sub-indicators evaluating the RACF’s policies, procedures and guidelines

Indicator	Numerator	Denominator	Target
Calculation of summary indicator			
Percentage of recommended and current RACF policies, procedures and guidelines	Number of recommended and current RACF’s documents supporting medication management processes/activities	10 (total number of statements)	High (towards 100%)
Sub-indicators regarding recommended and current RACF’s policies, procedures and/or guidelines		Possible responses	
1. The collection and review of feedback from the resident, carers, family and/or substitute decision-makers regarding medicine use		Available: Yes/No Current: Yes/No/NA*	
2. The provision of written medicines-related information to the resident, their carers, family and/or substitute decision-makers as part of any clinical consultation		Available: Yes/No Current: Yes/No/NA*	
3. The residents’ use of complementary and self-selected non-prescription medicines		Available: Yes/No Current: Yes/No/NA*	
4. A list of approved non-prescription nurse-initiated medications		Available: Yes/No Current: Yes/No/NA*	
5. The documentation of medication incidents and management of potential or actual harm arising from the medication incidents		Available: Yes/No Current: Yes/No/NA*	
6. Obtaining a best possible medication history (BPMH)		Available: Yes/No Current: Yes/No/NA*	
7. The roles and responsibilities of healthcare professionals regarding the use and review of medication chart		Available: Yes/No Current: Yes/No/NA*	
8. The continuity of medicines supply for all residents receiving care		Available: Yes/No Current: Yes/No/NA*	
9. The appropriate storage of all medications requiring refrigeration		Available: Yes/No Current: Yes/No/NA*	
10. The appropriate storage of all Schedule Eight (S8) medications		Available: Yes/No Current: Yes/No/NA*	

^*^NA is selected if the RACF does not have the recommended policy, procedure or guideline available. A score of zero is allocated for a NA response

*BPMH* best possible medication history, *NA* not applicable, *RACF* residential aged care facility, *S8* schedule eight medication

**Table 5:** Domain 3 Indicators and sub-indicators evaluating the RACF’s admission process

Indicators	Numerator	Denominator	Target
Calculation of summary indicator			
Percentage of newly admitted residents who have received recommended medicine-related admission processes	Number of residents admitted within the last 3 months with recommended medicine-related admission processes	Number of residents admitted within the last 3 months	High (towards 100%)
Sub-indicators for activities or processes documented at admission			
1. Percentage of newly admitted residents who have their medicine needs, preferences and medicine-taking behaviours documented	Number of residents admitted within the last three months who have their medicine needs, preferences and medicine-taking behaviours documented at admission	Number of residents admitted within the last 3 months	High (towards 100%)
2. Percentage of newly admitted residents wishing to self-administer medicines who were assessed for their capability to self-administer medications	Number of residents admitted within the last 3 months wishing to self-administer any of their medications who have their ability to self-administer these medication(s) assessed	Number of residents admitted within the last 3 months who wish to self-administer any of their medications	High (towards 100%)
3. Percentage of newly admitted residents with swallowing difficulties whose oral medications within their medication regimen have been assessed for appropriateness	Number of residents admitted within the last 3 months with swallowing difficulties who have the oral medications within their medication egimens assessed for appropriateness	Number of residents admitted within the last 3 months with swallowing difficulties	High (towards 100%)
4. Percentage of newly admitted residents with their vaccination status documented	Number of residents admitted within the last 3 months who had their vaccination status for influenza and COVID-19 documented	Number of residents admitted within the last 3 months	High (towards 100%)
5. Percentage of newly admitted residents who have nominated a person responsible for medicines-related decisions	Number of residents admitted within the last 3 months who have a nominated person responsible for medicines-related decisions documented	Number of residents admitted within the last 3 months	High (towards 100%)

*RACF* residential aged care facility

**Table 6:** Domain 4 Indicators evaluating the RACF’s medication review processes

Indicators	Numerator	Denominator	Target
Calculation of primary indicator			
1.Percentage of residents who have received a CMR by an appropriately qualified pharmacist within the last year	Number of residents who have received a CMR by an appropriately qualified pharmacist within the last 12 months	All current non-respite RACF residents	High (towards 100%)
Calculation of secondary indicators			
2. Percentage of newly admitted residents who have received a CMR by an appropriately qualified pharmacist since their RACF admission	Number of RACF residents admitted within the last 3 months who have received a CMR by an appropriately qualified pharmacist since their RACF admission	All newly admitted non-respite RACF residents	High (towards 100%)
3. Percentage of residents with polypharmacy who have received a CMR by an appropriately qualified pharmacist within the last year	Number of residents prescribed nine or more medications as per NQIP audit and who have received a CMR by an appropriately qualified pharmacist within the last 12 months	All non-respite RACF residents with polypharmacy	High (towards 100%)
4. Percentage of residents receiving an antipsychotic medication who have received a CMR by an appropriately qualified pharmacist within the last year	Number of residents who received an antipsychotic medication as per NQIP audit and have received a CMR by an appropriately qualified pharmacist within the last 12 months	All non-respite RACF residents receiving an antipsychotic	High (towards 100%)

*CMR* comprehensive medication review, *NQIP* national quality indicator program, *RACF* residential aged care facility

Data required for the indicators will be extracted from the RACF’s existing clinical information systems, including National Aged Care Mandatory Quality Indicator Program records, psychotropic medicines register, nursing progress notes, medical records, RACFs register of policies/procedures/guidelines, register of residents who have a completed comprehensive medication management review by a pharmacist, register of residents admitted to the RACF and MAC meeting agenda, minutes, and associated papers. The identifiable resident-level data required to complete the indicators will only be accessed by appropriate staff employed/contracted by the aged care provider organisation and by the knowledge broker dyad to assist with intervention delivery. Trial investigators will not have access to these data sources. Microsoft Excel-based ‘Guiding Principles Concordance’ data collection tools [38] were developed for the trial to assist data collectors in calculating RACF-level concordance for each trial indicator and sub-indicator. RACF-level concordance will be entered into REDCap. Aggregated data on Guiding Principles concordance for all participating RACFs will be reported at quarterly MEGA-MAC meetings and in the MEGA-MAC newsletters.

### RACF-level proportions of hospitalisation, quality of life, consumer experience, and medication-related incidents

Data on RACF-level proportions of hospitalisation, quality of life, and consumer experience will be extracted from the mandatory National Aged Care Quality Indicator Program records [39]. The following definitions will be used in line with the National Aged Care Quality Indicator Program:

- Hospitalisation: RACF-level proportion of residents hospitalised compared to the previous quarter. Hospitalisation will include emergency department presentations and hospital admissions within the last 3 months.
- Quality of life: RACF-level proportion of residents who report ‘excellent’ or ‘good’ quality of life as assessed using The Quality of Life Aged Care Consumers (QOL-ACC) tool compared to the previous quarter [40].
- Consumer experience: RACF-level proportion of residents who report ‘excellent’, or ‘good’ consumer experience using The Quality of Care Experience Aged Care Consumers (QCE-ACC) compared to the previous quarter.

Data on RACF-level prevalence of medication-related incidents will be extracted from each RACF’s electronic risk management system quarterly.

### RACF and MAC characteristics

Data on RACF-level characteristics will include: location (e.g. State, Territory), region (e.g. metropolitan, regional, rural, remote), number of residents, number of beds, number of residents who are female, mean resident age, number of health and care professionals employed/contracted and the health professional disciplines that typically visit the RACF weekly. Data on MAC characteristics will include: number of RACFs and residents the MAC governs, number of MAC members, discipline of each MAC member (e.g. general practitioner, nurse, pharmacist) and the discipline of the MAC chair. RACF characteristics will be collected at baseline. MAC characteristics will be collected at baseline and 9 months.

### Knowledge broker and MEGA-MAC Collaborative demographics

Demographic data will be collected from each knowledge broker dyad and MEGA-MAC collaborative panel member at time of recruitment. Knowledge broker demographics will include: gender, discipline (e.g. general practitioner, registered nurse, pharmacist), professional background (education, qualifications, practice experience, aged care experience) and length of time employed or contracted at the RACF. MEGA-MAC collaborative panel member demographics will include: gender, discipline (e.g. general practitioner, registered nurse, pharmacist), professional background (education, qualifications, practice experience, aged care experience, current role/s) and consumer roles.

### Knowledge broker dyad activities

Data from local action plans (including goals, type of local activities implemented, resources used, timeline, actual implementation, reflections) and MEGA-MAC meetings will be collected throughout the trial. Semi-structured interviews with knowledge broker dyads will also be conducted at 3 months, 6 months and at the conclusion of the trial to understand the delivery of the intervention at each RACF. Semi-structured interviews may also be conducted with other stakeholders at the conclusion of the trial. Optional anonymous online surveys will be conducted at the conclusion of each MEGA-MAC meeting to understand attendees’ perspectives on the meeting (e.g. aspects of the meeting that were useful, aspects that could be improved).

### Economic evaluation

Data for economic evaluation will include interventions costs (e.g. how much the intervention costs to be delivered), downstream costs (e.g. staff costs to attend MAC meetings), and costs of medication incidents and hospitalisations. Individual resident level data will not be collected, only RACF-level data. Economic data will be embedded within other trial data collection tools to minimise data collection burden where possible (refer to Appendix 2 for full details).

## Outcomes

### Primary outcome

The primary outcome will be the change in RACF-level concordance with the Guiding Principles post-intervention compared to pre-intervention. Concordance with the Guiding Principles will be measured as a score out of 28. This score will be composed of 19 indicators measured dichotomously (no = 0, yes = 1) and 9 indicators measured continuously (proportion between 0 and 1) (Tables 3, 4, 5 and 6). Higher scores will indicate greater concordance with the Guiding Principles.

### Secondary outcomes

Secondary outcomes will include change in the following outcomes post-intervention compared to pre-intervention:

- RACF-level concordance with trial indicators in each of the four key domains: 1) MAC meeting (Table 3); 2) policies, procedures and guidelines (Table 4); 3) admission processes (Table 5); and 4) medication review processes (Table 6).
- RACF-level concordance with each of the 28 individual sub-indicators
- RACF-level proportion of residents with one or more emergency department presentations or hospital admission. Proportion will be calculated using the total number of care recipients assessed for hospitalisation as a denominator.
- RACF-level proportion of residents who report ‘excellent’ or ‘good’ quality of life. Proportion will be calculated using the total number of care recipients who completed the quality of life assessment as a denominator.
- RACF-level proportion of residents who report ‘excellent’ or ‘good’ consumer experience. Proportion will be calculated using the total number of care recipients who completed the consumer experience assessment as a denominator.
- RACF-level total number of medication incidents reported in the previous three months.

### Sample size and power

A sample size of 15 RACFs provides 86% power to detect a standardised effect size of 0.5 in change between pre-intervention and post-intervention period measures. This is under the assumptions that an analysis approach at the margins incorporating all pre- and post- intervention measures will be employed, that there will be the correlation across the three pre-intervention within sites of *r* = 0.5 and the correlation across the three post-intervention measures within sites of *r* = 0.25.

## Analysis plan

### Effectiveness analysis

The primary analysis will employ a linear mixed model approach to investigate the effect of commencement of the intervention on the composite primary outcome. It will include treatment period (pre-intervention vs post-intervention) as a fixed, binary factor in the model; trial month as a continuous, fixed factor; and individual site as a random effect nested within the random effect of a MAC. An additional analysis will be presented that is the same as that described above but includes an interaction term between the treatment period and trial month factors. This is important as: i) there may be a natural underlying trend for facilities to increase in concordance with the Guiding Principles during the control period as a result of other background quality improvement processes; and ii) the intervention may not have an immediate effect on outcome domains being measured, but one that grows over time. We therefore have a conditional hypothesis that needs to be considered concurrently with our primary hypothesis to aid appropriate interpretation of results. This problem is analogous to that of interpretation of main effects and interaction effects arising from factorial trials. We will therefore follow recommendations for presentation of results of trials of this nature [41], and present both the models with and without the interaction term to allow comparison between the two.

Exploratory analyses to investigate the heterogeneity in treatment effects within sites and MACs will be undertaken by examining best linear unbiased predictions (BLUPs). This exploratory step is valuable, as an intervention that has highly variable impacts can be triangulated with our process evaluation to understand why the intervention may have had greater effect at some sites than others. This calculation will help to understand potential variation. Multiple imputation will be used to impute missing outcome values, where necessary.

All primary analyses will be conducted using the intention-to-treat (ITT) principle. Per protocol analysis will also be undertaken with the per protocol set including all RACFs without a major protocol deviation. Major protocol deviations may include, but are not limited to, the following:

- Loss of knowledge broker dyad (one or both members of the dyad); and
- Incomplete delivery of the intervention (e.g. local action plans, attendance at MEGA-MAC meetings).

Data will be analysed using Stata (StataCorp, College Station, TX), SAS (SAS Institute, Cary, NC) and the Statistical Package for the Social Sciences (SPSS, Inc., Chicago, IL).

### Economic analysis

A net benefit analysis will be conducted to examine the relative costs and benefits of implementing the intervention. We will model the results of the economic evaluation over several perspectives, including the perspective of the RACF, the Australian government as the funder of health and aged care and from a societal perspective. With each of these perspectives, we will vary the assumptions of who would be the payer of the program, with one set of models looking at if the RACFs was paying for the program directly, and another where the government would pay.

Modelling of cost data is required in this evaluation and economic assumptions will be made. Costs will include the direct costs of the intervention (e.g. salary costs of the knowledge broker) and downstream costs (e.g. staff time preparing for MAC meetings). Benefits will consider the monetised impact of hospitalisations and medication incidents. Measurement and valuation of activity will either be collected internally from the trial budget (e.g. salary costs of the knowledge broker) or valued using market rates (e.g. national or state-based awards as relevant) or by extrapolation based on previously published work for costs from external sources (e.g. the cost of staff to attend a MAC). The mean (Standard Deviation) will be presented for the cost of the intervention, downstream costs, cost of medication incidents and hospitalisations. To overcome variability in resident numbers at each of the RACFs included in the trial, the cost of the intervention, downstream costs, and costs of medication incidents and hospitalisations will be presented per bed at the RACF. Cost data will be presented in Australian dollars (AUD). Costs will be inflated accordingly by consumer price index (CPI) and presented as a consistent base year (e.g. 2025/2026 financial year).

### Process evaluation

A mixed method process evaluation will be undertaken using the Consolidated Framework for Implementation Research (CFIR) [42, 43]. Process evaluation will be performed through an analysis of qualitative data (e.g. local action plans, goals for improvement, interviews, anonymous MEGA-MAC meeting online surveys) and quantitative measures of engagement with the intervention (e.g. attendance to MEGA-MAC meetings, percentage of training completed by knowledge broker dyads). Data will be de-identified and qualitatively analysed to inform on outcomes relating to local process evaluations, barriers and enablers to change and feasibility and acceptability of the MEGA-MAC model for guideline implementation.

**Dissemination.**

Findings will be disseminated through lay summaries, conference presentations, and peer-reviewed publications in open access journals. The lay summaries will be developed in partnership with the three participating aged care provider organisations and the Project Interest Holder Group. All participating RACFs, MACs, and aged care provider organisations will be provided a final report summarising key findings. Research findings will also be disseminated through member organisations of the MEGA-MAC Project Interest Holder Group and their professional networks.

## Discussion

The MEGA-MAC trial investigates a novel system-level role for onsite aged care pharmacists to support close working relationships with their RACF and MAC to drive local collaborative medication-related quality improvement initiatives. The study will establish a national real-time clinical network for onsite aged care pharmacists and MAC representatives to share experiences and support residential aged care provider organisations to maximise the function of their MACs and implementation of the Guiding Principles into their RACFs. This study directly addresses national and international priority areas and the need for new models of guideline implementation to promote safe and effective medication use.

This trial will be the first to evaluate the novel role of a knowledge broker dyad to support guideline implementation in RACFs. The dyad aspect of the intervention makes this trial distinct from previous research [25] and was chosen to ensure the dyad would collectively have a strong understanding of the evidence being translated (the Guiding Principles – pharmacist), and a strong understanding of the context in which it is to be applied (the RACF – MAC representative). The inclusion of a pharmacist as part of the dyad is consistent with the Royal Commission into Aged Care Quality and Safety’s recommendation for pharmacists to have greater involvement with RACFs and the Australian Government funding for onsite aged care pharmacists [24, 44]. Clinical governance and quality use of medication are two of the five key roles of the new aged care onsite pharmacist model that commenced in July 2024 [45]. This trial will build evidence as to how pharmacists can work collaboratively in a system-level capacity. The MAC representative in the dyad brings an understanding of the RACF-, MAC- and aged care provider organisation-level context. They will facilitate organisational support and effective collaboration within interprofessional teams which are known facilitators associated with the success of embedded on-site aged care pharmacists [24, 46, 47]. Additionally, the national quality improvement collaborative builds on evidence of success in other settings [16, 20] and provides peer and expert support to address known challenges for health care professionals in RACFs such as ‘isolation’, inconsistent practices and lack of sharing of best-practice [12, 24, 46].

Knowledge brokers utilise a series of evidence-based knowledge translation strategies to support guideline implementation including staff training, audit and feedback and local action planning [15, 47]. The ability of the knowledge broker intervention to adapt to the local context is a key strength, however, gaining insight into the specific activities knowledge brokers undertake is essential for informing future scale-up efforts. The MEGA-MAC trial will generate important evidence on the roles of a knowledge broker through a comprehensive process evaluation guided by CFIR, while also assessing cost-effectiveness using a net-benefit analysis. This will enable resource modelling and projections and support the potential expansion and broader implementation of knowledge brokers in RACFs.

The MEGA-MAC intervention represents a transformative, system-level multi-faceted knowledge translation strategy that will generate critical insights on the role of knowledge brokers, MACs and quality improvement collaboratives for supporting guideline implementation and medication management in RACFs. The findings from this trial have the potential to inform future policy, define new roles for healthcare professionals, drive sustainable improvements in medication safety, and serve as a model for enhancing clinical governance in aged care and beyond.

## Supplementary Information

Appendix 1

Appendix 2

## Data Availability

Not applicable.

## Abbreviations

AHPRA: Australian Health Practitioner Regulation Agency
ANZCTR: Australian and New Zealand Clinical Trial Registry
DCT: Data collection tool
KB: Knowledge broker
IHI: Institute for Healthcare Improvement
MAC: Medication advisory committee
MEGA-MAC: Maximising Embedded Pharmacists in Aged Care Medication Advisory Committees
NHMRC: National Health and Medical Research Council
NSW TAG: New South Wales Therapeutic Advisory Group
NQIP: National Quality Indicator Program
PDSA: Plan, do, study, act
RACF: Residential aged care facility
REDCap: Research Electronic Data Capture
SMART: Specific, measurable, achievable, relevant, timely.
SPIRIT: Standard Protocol Items: Recommendations for Interventional Trials
QCE-ACC: Quality of Care Experience Aged Care Consumers
QoL: Quality of Life
QOL-ACC: The Quality of Life Aged Care Consumers
WHO: World Health Organization
