# Supplementary material for: Maximising Embedded Pharmacists in AGed CAre Medication Advisory Committees (MEGA-MAC): protocol for implementing Australia’s new guiding principles for medication management in residential aged care facilities using knowledge brokers and a national quality improvement collaborative
Source: Implement Sci. 2025 Aug 4;20:36. doi: 10.1186/s13012-025-01449-0 (PMC12323118; doi:10.1186/s13012-025-01449-0)
Supplement: Supplementary file 2 — Appendix 2 [file 13012_2025_1449_MOESM2_ESM.docx]

**Appendix 2: Data collection for economic evaluation**

| **Data required for economic evaluation** | **Data source** | **Time points** (months) | | | | | | | | | | |
| --- | --- | --- | --- | --- | --- | --- | --- | --- | --- | --- | --- | --- |
|  |  | *-6* | *-3* | *0* | | *3* | | *6* | | *9* | |  |
| ***Intervention costs*** | | | | | | | | | | | | |
| Cost of knowledge broker | Actual costs of knowledge broker will be provided from the trial budget. (Pharmacist at 4.5 hours per week per RACF for 9 months) |  |  | |  | |  | |  | | x | |
| Cost of MAC representative | Actual costs of MAC representative will be provided from the trial budget. (1.5 hours per week per RACF for the 9-month intervention period) |  |  | |  | |  | |  | | x | |
| Cost of the MEGA-MAC collaborative | Data will be collected from the trial budget and MEGA-MAC meetings to model costs, in line with economic assumptions. Frequency, duration and attendees to MEGA-MAC quarterly meetings will be determined from meeting minutes collected by researchers as part of the trial. A nominal amount will be presented for the costs associated with the preparation and dissemination of the quarterly MEGA-MAC newsletters. |  |  | |  | |  | |  | | x | |
| Online training for intervention | Actual costs of training for the knowledge broker will be provided from the trial budget. |  |  | |  | |  | |  | | x | |
| ***Downstream costs – staff costs for the following activities;*** | | | | | | | | | | | | |
| Attendance at RACF MAC meeting | RACF staff via survey. Staff costs will be modelled based on economic assumptions. | x | x | | x | | x | | x | | x | |
| Preparing the RACF’s report of residents’ experiences from RACF residents, their carers, family and/or substitute decision-makers regarding medication-related issues for MAC meeting. | Economic evaluation will use data captured as part of MEGA-MAC indicator 1 to model staff costs based on if this report was presented during the MAC meeting. | x | x | | x | | x | | x | | x | |
| Preparing the RACF’s report of RACF storage of medicines for MAC meeting. | Economic evaluation will use data captured as part of MEGA-MAC indicator 1 to model staff costs based on if this report was presented during the MAC meeting. | x | x | | x | | x | | x | | x | |
| Preparing the RACF’s report of medication incidents for MAC meeting. | Economic evaluation will use data captured as part of MEGA-MAC indicator 1 to model staff costs based on if this report was presented during the MAC meeting. | x | x | | x | | x | | x | | x | |
| Preparing the RACF’s report of trends in the percentage (or numbers) of residents with polypharmacy for MAC meeting. | Economic evaluation will use data captured as part of MEGA-MAC indicator 1 to model staff costs based on if this report was presented during the MAC meeting. | x | x | | x | | x | | x | | x | |
| Preparing RACF’s report of trends in the percentage (or n umbers) of residents who received an antipsychotic medication for MAC meeting. | Economic evaluation will use data captured as part of MEGA-MAC indicator 1 to model staff costs based on if this report was presented during the MAC meeting. | x | x | | x | | x | | x | | x | |
| Preparing the RACF’s report of trends in the percentage (or numbers) of residents who received a CMR from an appropriately qualified pharmacist for MAC meeting. | Economic evaluation will use data captured as part of MEGA-MAC indicator 1 model staff costs based on if this report was presented during the MAC meeting. | x | x | | x | | x | | x | | x | |
| Actioning items (i.e. follow up) after the MAC meeting. | Survey questions to determine time (in minutes) spent on follow up, by whom and items actioned. Staff costs will be modelled based on economic assumptions. | x | x | | x | | x | | x | | x | |
| Updating RACF’s Policies, Procedures and Guidelines captured in Domain 2. | Survey questions. |  |  | | x | |  | |  | | x | |
| Determining resident’s medicine needs, preferences and medicine-taking behaviours documented within two weeks of admission to the RACF. | Economic evaluation will use data captured as part of MEGA-MAC indicator 3 to model staff costs based on how many newly admitted residents that met this criterion. | x | x | | x | | x | | x | | x | |
| Determining capability of newly admitted residents’ ability to self-administer medicines wishing to self-administer one or more of their medicines. | Economic evaluation will use data captured as part of MEGA-MAC indicator 3 to model staff costs based on how many newly admitted residents that met this criterion. | x | x | | x | | x | | x | | x | |
| Assessing appropriateness of oral medication regimen for newly admitted residents with swallowing difficulties. | Economic evaluation will use data captured as part of MEGA-MAC indicator 3 to model staff costs based on how many newly admitted residents that met this criterion. | x | x | | x | | x | | x | | x | |
| ***Downstream costs - other*** |  |  |  | |  | |  | |  | |  | |
| Costs of CMR | Embedded additional question after MEGA-MAC Indicator 4 to determine how many residents received a CMR in the last 3 months to model costs. | x | x | | x | | x | | x | | x | |
| *Cost of hospitalisations* | Embedded additional question after NQIP Hospitalisations to model costs of hospital admissions based on the number of residents presenting to hospital, including ED, and the length of stay of admission. | x | x | | x | | x | | x | | x | |
| *Costs medication incidents* |  |  |  | |  | |  | |  | |  | |
|  | Embedded additional question after NQIP Consumer experience to model costs of medication incidents. | x | x | | x | | x | | x | | x | |
| *Date required for economic modelling* | | | | | | | | | | | | |
| MAC | Data will be collected from each MAC to model costs, as per economic assumptions. A separate data collection tool will be requested to be completed by each individual MAC, in addition to trial demographic data collection. |  |  | | x | |  | |  | | x | |
| RACFs | Data will be collected from each RACF to model costs, as per assumptions. A separate data collection tool will be requested to be completed by individual RACFs, in addition to trial demographic data collection. |  |  | | x | |  | |  | | x | |

Abbreviations; CMR, comprehensive medication review; DCT, data collection tool; ED, emergency department; MAC, medication advisory committee; MEGA-MAC, national quality improvement collaborative; NQIP, National Quality Indicator Program; RACF, residential aged care facility
